# TileQC: A system for tile-based quality control of Solexa data

**DOI:** 10.1186/1471-2105-9-250

**Published:** 2008-05-28

**Authors:** Peter C Dolan, Dee R Denver

**Affiliations:** 1 Department of Zoology and Center for Genome Research and Biocomputing, Oregon State University, Corvallis, Oregon 97331, USA

## Abstract

**Background:**

Next-generation DNA sequencing technologies such as Illumina's Solexa platform and Roche's 454 approach provide new avenues for investigating genome-scale questions. However, they also present novel analytical challenges that must be met for their effective application to biological questions.

**Results:**

Here we report the availability of *tileQC*, a tile-based quality control system for Solexa data written in the R language. *TileQC *provides a means of recognizing bias and error in Solexa output by graphically representing data generated by flow cell tiles. The data represented in the images is then made available in the R environment for further analysis and automation of error detection.

**Conclusion:**

*TileQC *offers a highly adaptable and powerful tool for the quality control of Solexa-based DNA sequence data.

## Background

New high-throughput sequencing technologies have arisen over the last decade that produce very large numbers of small sequencing reads (hundreds of thousands to millions), making possible the rapid and inexpensive sequencing and resequencing of genomes [1]. Despite the excitement generated by these new technologies, they also present substantial challenges that include sequence assembly of millions of short-read fragments (~30 bp for Illumina's Solexa sequencing approach) for de novo sequencing applications [2,3], and the rapid and accurate mapping of short sequence reads to genomic locations for resequencing [4]. Regardless of the application, one major concern is the ability to effectively characterize the reliabilities of DNA sequence reads deriving from "next-generation" platforms that rely on novel sequencing chemistries such as Solexa's reversible dye-labeled terminator approach. Furthermore, these platforms have abandoned the electrophoresis-based approaches of traditional Sanger sequencing; instead, DNA sequence data is collected in real-time from novel sequencing substrates. Development of quality-control tools for these next-generation DNA sequencing technologies is critical for their effective and accurate application to biological questions.

Illumina's Solexa sequencing approach consists of a process whereby DNA samples are nebulized to small pieces (~150 bp), then ligated to adapters that bind to linker molecules on the surface of a flow cell where amplified DNA clusters are ultimately sequenced in real-time using Solexa's reversible dye terminator approach [5]. Each flow cell contains eight lanes onto which DNA molecules from distinct samples can be independently sequenced. Each lane is subdivided into hundreds of tiles (200 tiles in earlier systems, 300 in the most recent system) – four images are collected from each tile (one for each of the four base dyes) per sequencing cycle. These tile images constitute the raw data from which DNA sequence information is ultimately derived. Illumina provides a standard front-end analysis pipeline for Solexa data where image analysis is carried out by Firecrest and base calls are made by Bustard. In making a base call, Bustard assigns a quality score (Q-score) to each of the 4 potential nucleotides. These Solexa quality scores range from -40 to 40. They are not equal to Phred quality scores, but are asymptotically identical [10]. Assuming no ambiguity, the nucleotide with the highest Q-score is called. In an ideal call, there is one +40 and three -40 s. The aggregate quality score (Q_AG_-score) for a base call is the maximum Q-score minus the sum of the remaining three Q-scores.

After Firecrest and Bustard, Eland provides alignments of individual Solexa sequence reads to a user-defined reference genome. Eland subdivides all sequence reads into eight categories: those where sequences align to unique genomic regions with 0, 1 or 2 mismatches (U0, U1 and U2, respectively), those where sequences align to repetitive regions with 0, 1 or 2 mismatches (R0, R1 and R2, respectively), those where there are three or more mismatches to the reference genome which is defined as the "no match" (NM) category, and those containing two or more bases that were unable to be called (QC).

Here we provide an openly available software program, *tileQC*, for quality control of Solexa output. *TileQC *relies on the R programming environment and a mySQL database server configured for use by the *tileQC *program. Minor changes in the initialization script allow almost any SQL server to be used. Initial configuration is minimal but flexible enough that a gamut of security options is possible.

*TileQC *features both qualitative and quantitative error detection. The qualitatively oriented functions display the locations of reads on a tile as dots in a square. The read's color and size are coded using Eland categorizations and/or the Q_AG_-score data derived from Bustard. The Eland-coded images represent the data after all other processing has occurred and reveal irregularities arising during any stage of the processing pipeline. Q_AG_-score coded images, on the other hand, are produced from the Bustard output and not only produce a greater range of values than the Eland categorizations, but also have greater resolution, allowing the Solexa output to be analyzed down to the level of individual read cycles. This increased flexibility may obscure errors that are obvious at the Eland level. However, once an error is detected, the Q_AG_-score coding allows for a more accurate assessment of that detected error's underlying cause and/or location.

The guiding philosophy behind *tileQC*'s qualitative error assessment features is that the researcher's visual pattern recognition is the best way to detect novel errors. Once a new type of error is identified the data extraction features of the program may then be used as a starting point for the programmatic detection and filtration of similar errors.

## Implementation

The current version (*tileQC *1.0, see Additional file 1) runs on Windows, Linux, and Macintosh operating systems, and requires the programming environment R (version 2.5 or higher) and a properly configured MySQL server (detailed directions for configuration are available at [8]). The package 'RMySQL' must also be installed within the R environment. (The package 'RMySQL' also requires the package 'DBI', however, installing 'RMySQL' will install the 'DBI' package automatically).

The R software is available for download [6] as is the 'RMySQL' package (see the FAQ at [6] for details on downloading and installing an R package). The database server MySQL is also available for free download [7]. The *TileQC *system was implemented using the R language: source code, installation instructions and tutorials are available at the *tileQC *website [8].

In order to convert text-files into database form (and/or import data directly from the text files) the utilities *sed*, *tr*, *grep*, and *wc *must also be installed. These programs are part of the standard installation on most flavors of Unix (including recent versions of the Macintosh OS). For the win32 platform all the necessary programs are included in GNU utilities for win32 available at [9].

## Results and Discussion

Throughout this section all Solexa data used in examples was generated from several of our *Caenorhabditis elegans *genomic DNA runs (unpublished data) on an Illumina Genome Analyzer. All *C. elegans *data were subject to the standard Solexa data analysis pipeline prior to application of *tileQC *tools.

The first role of *tileQC *is to facilitate the conversion of text based Solexa pipeline output to a more flexible SQL database format (in our case the MySQL database server). If a compatible database does not already exist, *tileQC *will (upon request) create one. Creating a database requires that both the SQL server and the *tileQC *program be properly configured (see [8] for details). Once the Eland and Q_AG_-score data is in database form the full power of both SQL and R may be brought to bear upon the analysis of that data. Encapsulating the database connection within an R object enables the mundane details to be managed invisibly and frees the researcher to focus on the analysis of the data rather than the mechanics of accessing and manipulating that data. Although supplementary to the package's primary purpose of tile-based quality control (QC), this feature is useful in its own right, and simplifies the mechanics of querying a database containing Solexa data. The standard SQL query language is enhanced by the inclusion of a simple form of expression substitution. Here, for example, we see the extraction of five reads covering the location 332,080 in Chromosome I of the *C. elegans *genome (note the use of #current.table# in the SQL command):

> celegans$runSQL("select seq, type, locus, muta, mutb from #current.table#

where locus >= 332048 and locus <= 332080 and segment = 'CHR_I' limit 5")

seq type locus muta mutb

1 AATTTTTTGAATTTGCTCGCCGCATTTCGACTTTCT U2 332053 23A 28T

2 TGAATTTGCTCGCCGAATTTCGACTTTCTTACAATT U2 332060 21T 30G

3 GAATTTGCTCGCCGAATTTCGACTTTCTGACAATTT U1 332061 20T

4 GAATTTGCTCGCCGAATTTCGCCTTTCTGACACTTG U2 332061 20T 22A

5 GAATTTGCTCGCCGAATTTCGACTTTCTTACAATTT U2 332061 20T 29G

The primary purpose of the package is, of course, tile-based quality control. Often there are patterns in the errors generated during the Solexa sequencing process that become visible when the physical locations of a tile's reads are plotted in colors and sizes that depend upon the category to which they have been assigned by Eland. For this purpose, the *tileQC *package contains functions that are optimized to create such qualitative displays. The visual representation appears on the left and a relative frequency histogram of the number of reads in each Eland category for that tile appears to the right. The researcher may select which categories of read are to be displayed, and even filter the unique reads based upon whether they match the forward strand, the reverse strand, or either. The homogeneity of the Solexa process ensures that, when the machine is functioning properly, the relative frequencies are similar from tile to tile and distributed uniformly across each tile. Major discrepancies in these conditions are immediately discerned by sight.

**Figure 1:**
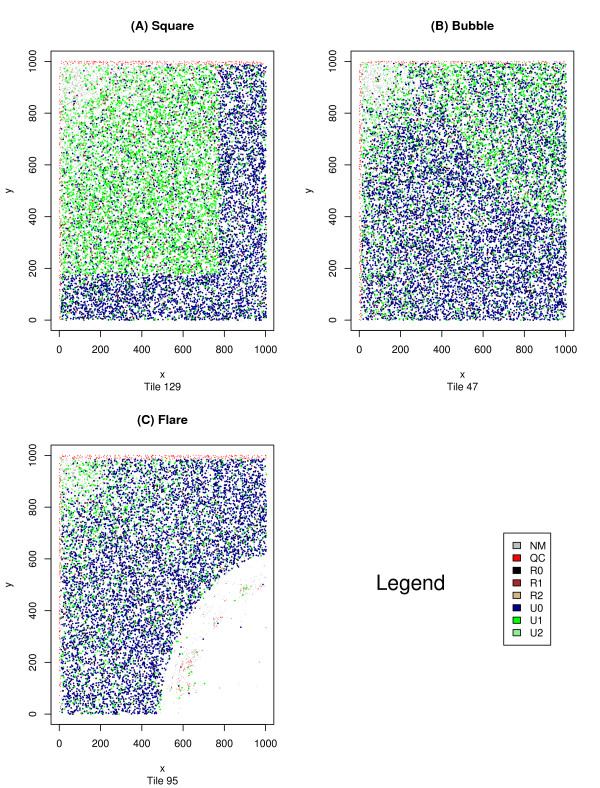
**Aberrant Solexa tiles**. This figure displays three distinct types of errors that we have seen occur on Solexa tiles. The image was generated using *plotQTile *with the color-by category option. The tile data was drawn from tiles 129, 85, and 6 respectively – all produced from the same lane and run. The error depicted in 1b appears to be caused by a bubble in one of the reagents. This bubble increased the error rate, converting U0 reads into U1 reads. From further investigation we know this occurred during cycle 28. The error depicted in 1c is an area in which no reads at all occurred – possibly a problem with the DNA binding to the flow cell, or a smudge on the surface of the flow cell. The error depicted in 1a remains enigmatic.

Many such discrepancies are small and their effects are limited to one, or (possibly) a few, tiles. Figure 1 contains examples of three such situations. Often these aberrations have obvious causes such as bubbles in the reagents. However some, such as the rectangle in Figure 1a, remain mysterious. Detection of aberrant tiles is particularly important for researchers doing single nucleotide polymorphism (SNP) detection as a single tile with an increased error rate may yield a variety of false positives.

**Figure 2:**
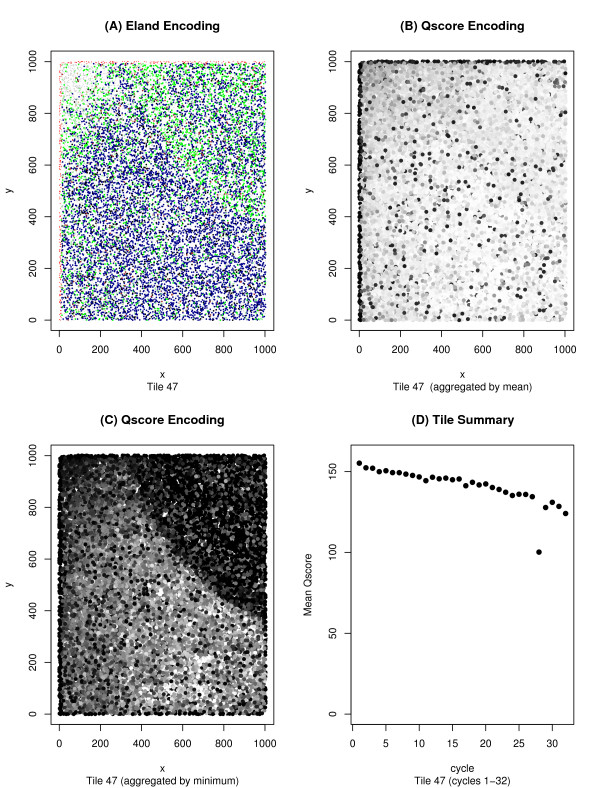
**Analysis of a single tile using *plotQTile *and *cycleplot***. Here we see three distinct views of tile 47 from Figure 1b. Similar to Figure 1, Figures 2a–2c were generated using the function *plotQTile*. Figure 2a uses the color-by category option to display the position of the reads color-coded according to their Eland category. In Figure 2b, the gray intensity values are generated by taking the mean across the first 32 cycles and then normalizing. Figure 2c is similar to 2b, but uses the minimum value across those 32 cycles instead of the mean. Figure 2c shows that some bubbles are visible from a Q_AG_-score perspective, but the contrast between 2b and 2c shows that one must be careful to choose the proper aggregating function. Figure 2d was generated using the *cycleplot *function. It displays the mean Q_AG_-score for cycles 1–32 on tile 47, and showcases the ability to detect the source of an error by decomposing the data according to cycle. Here we see the drop in average intensity that occurred during cycle 28.

**Figure 3:**
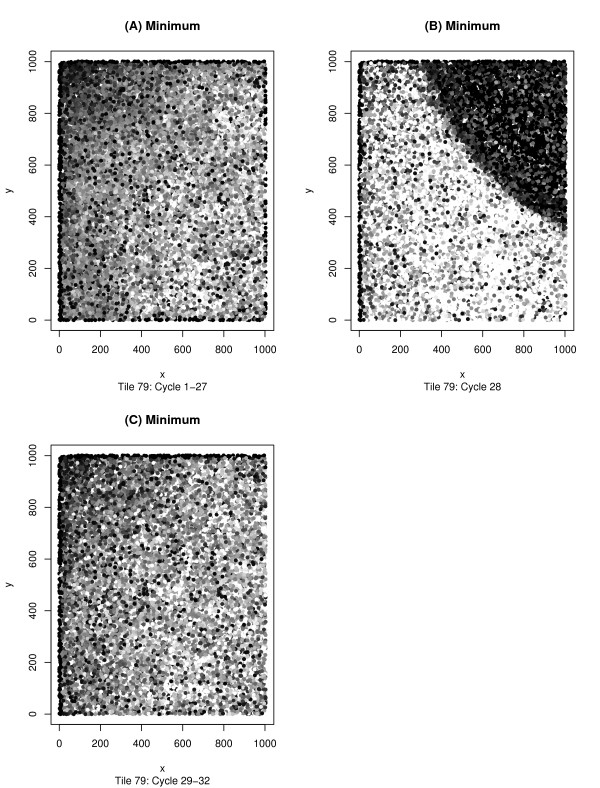
**Filtering the data by cycle**. This figure is a closer exploration of the bubble on tile 47 analyzed in Figure 2. The *tileQC *system allows the output of the *plotQTile *function to be filtered according to cycle. This graph was generated using *plotQTile *and the *cycles *option. Fig. 3a shows the minimum Q_AG_-score across cycles 1–27 by using *cycles = 1:27*, Fig. 3b restricts to the 28^th ^cycle by using the *cycle = 28 *option, and Fig. 3c restricts to cycles 28–32. From these 3 tiles it is clear that the problem behind the bubble occurred during cycle 28.

**Figure 4:**
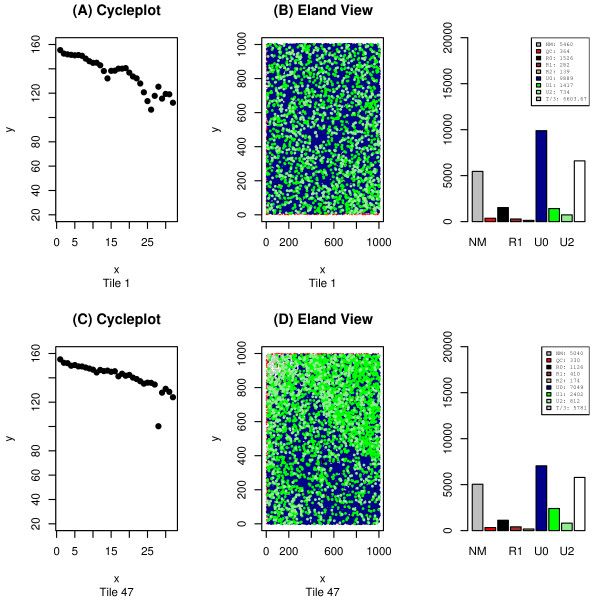
**An erratic tile with believable results**. This image was generated using both *plotTile*, and *cycleplot*. It shows two tiles. One of the tiles (tile 1) has the usual number of reads for a tile from that run, and a typical breakdown of those reads into the Eland categories. Note the number of reads in the U0 and U1 categories in the histogram on the top right. Nevertheless, as can be seen in Figure 4a this tile has widely varying differences amongst the mean intensities (per cycle). The other tile is the familiar tile 47. The intensity levels are much better behaved, except for the problem in cycle 28. But despite this fact, there is an elevation in the U1 levels on tile 47. This is particularly notable because the lowest intensity cycle on tile 47 is at roughly the same level as the lowest found on tile 1.

Tile-plots using Q_AG_-score data allow for a more in depth analysis of the data and a better identification of an error's source, but one must be cautious – many types of errors that are clear from the Eland perspective may be difficult to discern from the Q_AG_-score perspective. Sometimes, this can be alleviated by knowing the proper way to transform the Q_AG_-score data into intensity data. Any desired function may be applied to a read's set of Q_AG_-scores and the output of that function is normalized and transformed into an intensity value for the dot corresponding to that read. An example of this process can be seen in Figure 2 where we see three views of the same tile. The first, Figure 2a, uses Eland encoding, whereas Figures 2b and 2c use two distinct Q_AG_-score encodings. In Figure 2b we see the results of applying the function *mean *across the first 32-cycles of a read. The error detected through Eland categorization is invisible in this image, but once again is easily detectable in Figure 2c where the minimum of the 32 inter-read Q_AG_-scores are being plotted. The source of the error becomes apparent in Figure 2d – a *cycleplot *displaying the mean Q_AG_-score for the first 32 cycles of the aberrant tile. We see from this graph, that the problem arises during cycle 28, and Figure 3, showing the minimum Q_AG_-scores for a variety of cycles, confirms this interpretation. From this example, one might be tempted to assume that any tile with a cycle whose mean Q_AG_-score drops below a certain value is to be discarded, but it is possible for a non-aberrant tile to have mean Q_AG_-scores this low (as can be seen in Figure 4).

**Figure 5:**
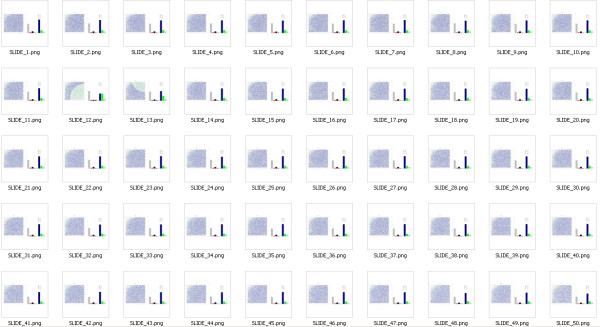
**Consistent errors across multiple tiles**. Here multiple tiles are shown. In each of the tiles, the upper left corner looks faded. That is due to an increase in error rate that causes reads to be categorized as NM. This is a global issue – spanning multiple tiles.

The visual pattern recognition of a researcher may also discern more subtle and global biases that escape simple numeric detection as in Figures 1, 2, 3, 4 where we see problematic reads occurring more frequently on the boundary of tiles, or in Figure 5 where a consistent increase in error rate is associated with reads from the upper left corner of a tile. Once such errors are known to occur, more sophisticated statistical techniques may be used to detect and remove the resulting biases, or at the very least to filter out the offending data.

**Figure 6:**
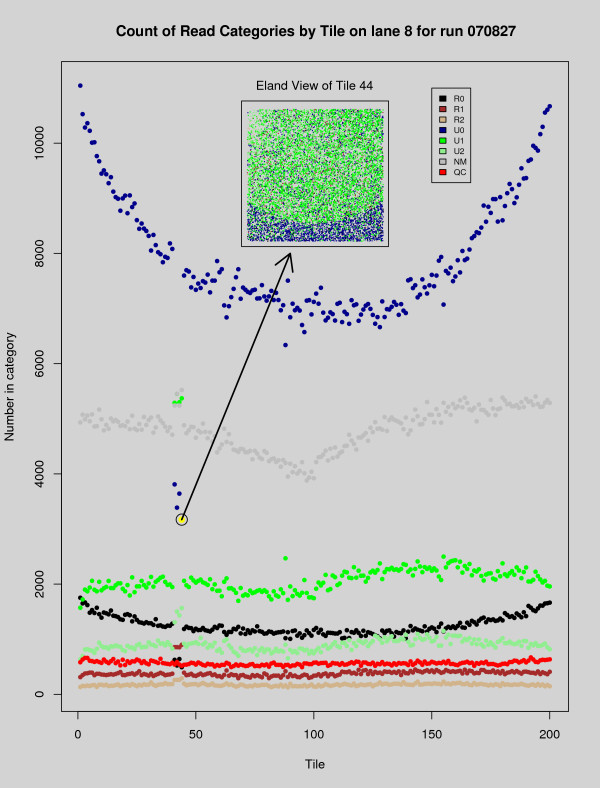
**Problems at the boundaries**. Here we see a typical tile (tile 44) superimposed upon a summarization plot. The tile graph was generated using *plotTile*, and the summarization using *plotSummary *with *summary = 2*. The overlap of the two graphs (and the arrow) were produced using R, but are not produced automatically by *tileQC*. The red dots on the top of the tile indicate reads for which Bustard was unable to make a base-call. The dots in the summarization graph denote the number of reads (per tile) in each of the Eland categories. Note the droop in the blue U0 dots.

Despite the power of the human visual system, some patterns of error that occur over an entire lane of Solexa data may not be apparent from direct observation of the 200–300 QC plots associated with that lane. However, some of these global biases are revealed when summarizing statistics are extracted for each tile and plotted in a single graph. In Figure 6 the blue dots correspond to the total number of perfect matches in each tile. The tiles within each lane are arranged as two vertical strips. In Figure 6, the left strip contains tiles 1–100 (with 1 at the top). The right strip contains tiles 101–200, but this time 101 is at the bottom and 200 at the top. The droop in the graph is indicative of an error rate that increased as the machine worked its way from the top of the lane to bottom. Aberrant tiles may also become visible from this perspective – in this same figure a tile with a particularly low number of unique matches was identified (the dot marked by yellow), and the graph of that tile's Eland categorized reads was superimposed upon a blank section of the summarization graph to illustrate how a bubbled tile may be detected in a summarization graph. We are presently working to improve the summary statistics features of *tileQC *and anticipate more advanced summary reports in future versions of this software.

## Conclusion

The *tileQC *system offers a versatile and powerful tool for the quality control of Solexa-based DNA sequence data. Future challenges include the development of an interface that unifies the task of summarization with that of quantitative testing. This short-term goal (partially completed) will lead to a plug-in style of summarization and analysis that will allow researchers to flexibly encapsulate any desired post-processing or data extraction within a shareable R object. Mid-range goals include an interactive graphical interface for more convenient data exploration as well as a freely available library of analytic modules.

## Availability and requirements

The *tileQC *system is freely available from [8]. It requires R (version 2.5 or higher), the R package 'RMySQL' and MySQL (version 5.0 or higher). In order to convert Solexa output from text to database form it requires the Solexa pipeline (up to version 0.3) output files of the form '_prb.txt' and '_eland_result.txt' as well as the utilities *wc, grep, tr*, and *sed*.

## Abbreviations

The abbreviations are Q_AG_-score: aggregate quality score; SNP: single nucleotide polymorphism.

## Authors' contributions

PCD contributed to writing the manuscript and developed the software. DRD contributed to writing the manuscript. Both authors have read and approved the final manuscript.
